# Time – The fourth dimension of immune cells

**DOI:** 10.1002/mco2.682

**Published:** 2024-08-04

**Authors:** Guiming Li, Wenjun Zhang, Jing Yang

**Affiliations:** ^1^ Department of Haematology Tongji Hospital Shanghai Key Laboratory of Signaling and Disease Research Frontier Science Center for Stem Cell Research School of Life Sciences and Technology Tongji University Shanghai China

**Keywords:** CAR‐T therapy, drug discovery, immunotherapy

## Abstract

Deciphering the intricate cell‐state transitions orchestrating immune adaptation over time stands as a cornerstone for advancing biological understanding. However, the lack of empirical in vivo genomic technologies capable of capturing cellular dynamics has posed a significant challenge. In response to this gap, a groundbreaking study introduces Zman‐seq, a single‐cell technology that records transcriptomic dynamics across time by incorporating time stamps into circulating immune cells, enabling their tracking in tissues for extended periods. The application of Zman‐seq in glioblastoma research has successfully unraveled the cell state and molecular trajectories underlying the dysfunctional immune microenvironment. Understanding the temporal aspects of cell‐state transitions during immune responses is pivotal for advancing our knowledge in biology. The emergence of Zman‐seq addresses the current limitations in empirical in vivo genomic technologies, offering a revolutionary approach to studying the dynamics of immune cells over time. This highlight comprehensively explores the implications of Zman‐seq in resolving cell‐state transitions and molecular trajectories within the dysfunctional immune microenvironment in different types of immunotherapy. This technique has particular potential for chimeric antigen receptor T‐cell therapy, overriding drug resistance, clinical medication optimization, and facilitating drug development. In particular, this article discusses potential strategies for improving the efficacy of clinical treatments.

## Graphical abstract

Potential implications of Zman‐seq in different types of clinical‐related therapies. Zman‐seq offers a more nuanced approach to modulating immune responses, especially in empowering chimeric antigen receptor T‐cell therapy, overriding drug resistance, optimizing clinical medication, and facilitating drug development, in either cancer or non‐cancer diseases.

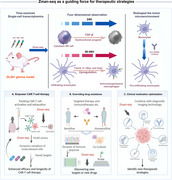

1

In the latest issue of the *Cell*, Zman‐seq was introduced as a novel methodology characterized by integrating time stamps into circulating immune cells, which allows the recording of transcriptomic dynamics over an extended period.^1^ This technology facilitates the tracking of individual cells within tissues for days, providing a unique temporal dimension to single‐cell analysis. Its broad applicability positions Zman‐seq as a transformative tool for empirically measuring differentiation trajectories.

The application of Zman‐seq in glioblastoma research has yielded groundbreaking insights into the temporal dynamics of immune responses. Within 24 h of tumor infiltration, cytotoxic natural killer (NK) cells exhibited a transition to a dysfunctional program regulated by transforming growth factor‐beta 1 signaling. Infiltrating monocytes, over a span of 36–48 h, differentiated into immunosuppressive macrophages marked by the upregulation of suppressive myeloid checkpoints Trem2, interleukin‐18 bp, and Arg1. A pivotal aspect of the study lies in the therapeutic interventions guided by Zman‐seq findings. The administration of an antagonistic anti‐TREM2 antibody successfully reshaped the tumor microenvironment by redirecting the monocyte trajectory toward pro‐inflammatory macrophages.^1^ This demonstrates the potential of Zman‐seq not only as an observational tool but also as a guiding force for therapeutic strategies. Zman‐seq offers a more nuanced approach to modulating immune responses, especially in chimeric antigen receptor (CAR) T‐cell therapy, drug resistance, clinical medication optimization, and drug development in cancer or non‐cancer diseases (Figure 1).

**FIGURE 1:**
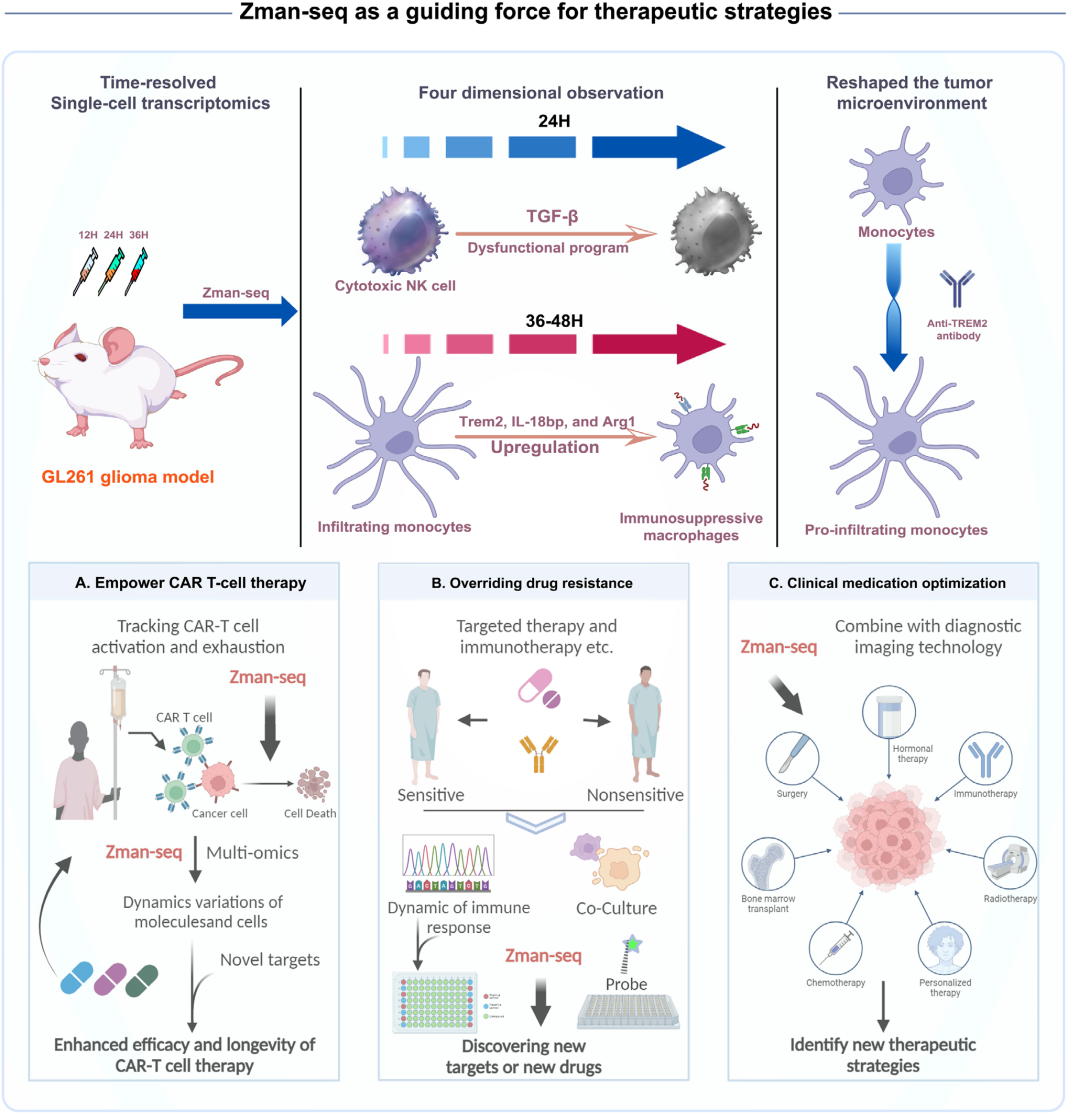
Potential implications of Zman‐seq in different types of clinical‐related therapies. Zman‐seq offers a more nuanced approach to modulating immune responses, especially in empowering chimeric antigen receptor (CAR) T‐cell therapy, overriding drug resistance, optimizing clinical medication, and facilitating drug development, in either cancer or non‐cancer diseases. Created with BioRender.com.

CAR‐T cell therapy has emerged as a cancer therapy with great potential, exhibiting effective antitumor activity in the treatment of hematological malignancies, especially in relapsed/refractory (R/R) B‐cell malignancies. However, there are significant challenges related to CAR‐T cell therapy that might be serious or even life‐threatening, such as CAR‐T‐related toxicities and resistance to CAR‐T cell therapy. Zman‐seq has demonstrated potential for CAR‐T therapy, as it allows for the detection of T cell activation and exhaustion states to varying degrees in cells sourced from patients and used for CAR‐T production, as well as CAR‐T cells administered back to patients. By employing this time‐related single‐cell technique together with ATAC‐seq or CUT&Tag to capture the dynamic chromatin accessibility landscape of CAR‐T cells, researchers can gain insights into the molecular dynamics of CAR‐T cells over time, ultimately understanding the temporal variations of molecules and cells within the tissue. This enables the identification of key molecular signals contributing to T‐cell exhaustion and the timing of their occurrence.

By combining spatial transcriptomics with time labeling as the fourth dimension, researchers can map the spatiotemporal dynamics of CAR‐T cells during expansion or exhaustion. This approach has allowed for a comprehensive analysis of the changes in gene expression patterns and cellular interactions during CAR‐T cell treatment. By tracking the dynamics of immune cells in the tumor microenvironment over time, for example, with anti‐CD45 labeling of the tumor‐infiltrating lymphocytes, factors can be identified that contribute to the development of CAR‐T cell exhaustion, modest anti‐tumor activity, and restricted trafficking, and thus may help to limit tumor infiltration and the incidence of severe life‐threatening toxicities.^2^ In addition, this approach can offer potential strategies to overcome these significant challenges.^2^

Innovative strategies and approaches to engineer more powerful CAR‐T cells, including CAR‐NKs, with improved anti‐tumor activity and decreased toxicity include combining Zman‐seq with CRISPR screening or small molecule screening in CAR‐T‐tumor coculture models in vitro or in vivo.^3^ By selectively analyzing the genes or small molecules that promote CAR‐T cell survival and functionality, while reducing the risk of host versus graft for CAR‐NKs, insights can be provided into potential strategies to enhance the efficacy and longevity of CAR‐T cell therapy. This approach holds promise for improving CAR‐T cell‐based immunotherapy by identifying novel targets and therapeutic interventions to overcome the challenges associated with CAR‐T cell exhaustion.

Another important application of Zman‐seq is overriding drug resistance. This new technology may provide valuable insights into the intricate dynamic between cancer treatment and the immune response associated with resistance to chemotherapy, radiotherapy, targeted therapy, and recently immunotherapy.^4^ It employs a powerful technique to comprehensively analyze the changes in immune cell populations and gene expression profiles between sensitive and nonsensitive patients. One notable observation is the depletion of specific subsets of immune cells, such as cytotoxic T cells and NK cells, following therapy. This depletion can compromise the anti‐tumor immune response, potentially leading to tumor evasion and disease progression. Furthermore, Zman‐seq may highlight the altered gene expression patterns in immune cells after treatments. These changes reflect potential dysregulation in immune signaling pathways, which may further influence the efficacy of immunotherapy and patient outcomes. This emphasizes the importance of considering the dynamic nature of the immune response during cancer treatment, which can guide the development of novel therapeutic strategies aimed at enhancing the effectiveness of immunotherapy and improving patient outcomes in the fight against cancer.

Using Zman‐seq to optimize the clinical medication of patients may yield significant insights including the impact of treatments, such as kinase inhibitors or immune checkpoint blockers on immune response within the tumor microenvironment.^5^ Through comprehensive analysis using Zman‐seq, notable changes in immune cell populations and gene expression profiles following different treatments can potentially be observed and this could conceivably lead to the identification of new anti‐tumor targets and new treatment paradigms. This underscores the importance of understanding the interplay between different drugs and immune cells in the context of tumor treatment. Such knowledge can inform the development of combination therapies that harness the synergistic effects of kinase inhibitors and immunotherapy, ultimately improving patient outcomes and advancing the field of cancer drug development. Moreover, during the process of medication optimization, Zman‐seq can be combined with imaging technology (such as light or fluorescence‐based barcode probes) to provide optimized therapeutic strategies for patients, instead of puncture surgery or sorting cells out with flow cytometry. In addition, it is also possible to use a real‐time fluorescence tracking technology in organoid 3D cultures, for example, a combination of the Live‐Cell analysis system with organoid‐drug co‐culture models, to facilitate research and drug screening applications with Zman‐seq or Xman‐seq (real‐time or other dimensions).

Zman‐seq can potentially be applied to other research areas, for example, infectious diseases, autoimmune diseases, drug development, and organ transplantation. With the ability to track the dynamic changes in different cells during the stages of infection, this approach aims to offer a deeper understanding of how the immune system attacks host tissues over time, and it also aims to provide a platform to assess the impact of candidate drugs on the dynamic states and functions of immune cells. In addition, by monitoring the trajectory of immune cells before or post‐transplantation, Zman‐seq could help to better understand specific biological mechanisms, pathways, and processes, and thus identify new therapeutic targets and develop novel targeted therapies or more effective drugs.

Zman‐seq emerges as a revolutionary technology, providing a temporal dimension to single‐cell analysis and unraveling the intricacies of immune cell dynamics over time. As we venture into an era of precision medicine, Zman‐seq stands as a beacon, guiding us toward a deeper comprehension of immune adaptation and offering novel avenues for therapeutic intervention.

## AUTHOR CONTRIBUTIONS

G.L. and J.Y. wrote the manuscript. J.Y. and W.Z. provided valuable scientific feedback. All authors have read and approved the final manuscript.

## CONFLICT OF INTEREST STATEMENT

The authors declare no conflict of interest.

## ETHICS STATEMENT

Not applicable.

## Data Availability

Data sharing is not applicable.
